# Relative abundance of short chain and polyunsaturated fatty acids in propionic acid-induced autistic features in rat pups as potential markers in autism

**DOI:** 10.1186/1476-511X-13-140

**Published:** 2014-08-31

**Authors:** Afaf El-Ansary, Laila Al-Ayadhi

**Affiliations:** Biochemistry Department, Science College, King Saud University, P.O box 22452, Zip code 11495 Riyadh, Saudi Arabia; Autism Research and Treatment Center, Riyadh, Saudi Arabia; Shaik AL-Amodi Autism Research Chair, King Saud University, Riyadh, Saudi Arabia; Department of Physiology, Faculty of Medicine, King Saud University, Riyadh, Saudi Arabia; Medicinal Chemistry Department, National Research Centre, Dokki, Cairo, Egypt

**Keywords:** Propionic acid, Rodent model, Autism, Short chain fatty acids, Polyunsaturated fatty acids, Relative values

## Abstract

**Background:**

Fatty acids are essential dietary nutrients, and one of their important roles is providing polyunsaturated fatty acids (PUFAs) for the growth and function of nervous tissue. Short chain fatty acids (SCFAs) are a group of compounds derived from the host microbiome that were recently linked to effects on the gut, the brain, and behavior. They are therefore linked to neurodevelopmental disorders such as autism. Reduced levels of PUFAs are associated with impairments in cognitive and behavioral performance, which are particularly important during brain development. Recent studies suggest that omega -3 fatty acids such as eicosapentaenoic acid (EPA) and docosahexaenoic acid (DHA) are involved in neurogenesis, neurotransmission, and protection from oxidative stress. Omega-3 PUFAs mediate some of these effects by antagonizing Omega-6 PUFA (arachidonic acid, AA)-induced proinflammatory prostaglandin E_2_; (PGE_2_) formation.

**Methods:**

In this work, the absolute and relative concentrations of propionic (PPA), butyric and acetic acids, as well as PUFAs and their precursors (α-Linolenic and linoleic), were measured in the brain tissue of PPA-neurointoxicated rat pups (receiving 250 mg PPA/Kg body weight for 3 consecutive days) as a rodent model with persistent autistic features compared with healthy controls.

**Results:**

The data revealed remarkably lower levels of omega6/omega3, α-Linolenic/Linoleic, α-Linolenic/EPA, α-Linolenic/DHA, EPA/DHA, and AA/Linoleic acid ratios in PPA-intoxicated rats. The role of these impaired ratios is discussed in relation to the activity of desaturases and elongases, which are the two enzymatic groups involved in the synthesis of PUFAs from their precursors. The relationship between the abnormal relative concentrations of the studied fatty acids and oxidative stress, neurotransmission, and neuroinflammation is also discussed in detail.

**Conclusions:**

This study demonstrates that fatty acid ratios are useful for understanding the mechanism of PPA neurotoxicity in a rodent model of autism. Therefore, it is possible to use these ratios for predictions in patients with this disorder.

## Introduction

Lipids constitute nearly 60 percent of the brain’s structure. Fatty acids are among the most important molecules that determine the brain’s integrity and ability to work efficiently. Clinical observational studies have linked fatty acid level imbalance to impaired brain performance and diseases such as autism, which is a neurodevelopmental disorder [1]. Physical signs consistent with fatty acid deficiency such as frequent urination, dull and dry skin and hair, and brittle nails have been linked to autism [1]. Additionally, essential fatty acids are important for brain development during both the fetal and postnatal period. Beyond their important role in building the brain’s structure, certain fatty acids act as messengers and are involved in the synthesis and function of neurotransmitters and the molecules of the immune system.

Neuronal membranes contain phospholipid pools that are reservoirs for the synthesis of specific lipid messengers following neuronal stimulation or injury. These messengers in turn participate in signaling cascades that can promote either neuronal injury or neuroprotection [2, 3].

Dietary essential fatty acids (EFAs) mediate brain function and structure during development and are involved in many brain-related disorders such as autism. Fatty acids are commonly classified as saturated, monounsaturated, or polyunsaturated (PUFA), depending on their chemical structure and chain length, which can vary from 12 to 26 carbon bonds. The two types of PUFAs include EFA, linoleic acid (LA: 18:2, n- 6) and α-linolenic acid (ALA: 18:3, n-3). The brain cannot distinguish between longer chain fatty acids that have been synthesized in the brain and those that have been obtained from diet and have crossed the blood–brain barrier. Clearly, the blood–brain barrier is key to the bioavailability of brain EFA and PUFAs.

The short chain fatty acids (SCFAs) acetate (C2), propionate (C3) and butyrate (C4) are the main metabolic products of anaerobic bacterial fermentation in the intestine. In addition to their important role as fuel for intestinal epithelial cells, SCFAs modulate different processes in the gastrointestinal (GI) tract such as electrolyte and water absorption. These fatty acids have been recognized as potential mediators of the effects of the gut microbiota on intestinal immune function and gut-brain axis interaction [4]. Recently it was reported that the three types of SCFAs (acetate, propionate, and butyrate) reduce the production of proinflammatory factors, including TNF-α, IL-1β, IL-6, and NO. Additionally, SCFAs enhance the production of the anti-inflammatory cytokine IL-10 in low concentrations (1–1,200 μmol/L) [5].

In spite of the protective effects of SCFAs, propionic acid (PPA) neurotoxicity was recently demonstrated via intraventricular direct infusion into rat brains [6], passage from the gut to the brain in the case of acute PPA orally administered to rat pups [7] or Chronic administration on postnatal days 5–28 [8] and, most recently, subcutaneous injection once a day (500 mg/kg) in pregnant rats on gestation days G12–16 [9].

This work is an attempt to understand the mechanism of PPA neurotoxicity by considering the impact of relatively impaired level SCFAs and PUFAs in PPA-intoxicated rats compared with a healthy untreated control group; the results suggest that the ratio is more important than the quantity of individual fatty acids.

## Material and methods

### Animals

The experimental assays for this study were performed on 16 young (approximately 21 days old) male western albino rats (45 to 60 g). Rats were obtained from the animal house of the pharmacy college at King Saud University and were randomly assigned to two groups of eight rats each. The first group of rats was given a neurotoxic oral dose of PA (250 mg/kg body weight/day for three days; n = eight) dissolved in 0.2 M phosphate buffered saline and was termed the oral buffered PA-treated group. The second group consisted of rats to which only phosphate buffered saline was administered and was used as a control group (n = eight). The two groups of rats were individually housed under controlled temperature (21 ± 1°C) with ad libitum access to food and water. The protocol was approved by the Ethics Committee of the King Saud University, and all experiments were performed according to the guidelines of the National Animal Care and Use Committee.

### Fatty acid profiling

Lipids were extracted from the brain tissue, and the fatty acids were methylated using 3 N methanolic HCL in sealed vials under nitrogen and incubated at 100°C for 45 min. The methyl esters of the free fatty acids were extracted with hexane, and the fatty acid composition of the extract was analyzed with a gas chromatograph (Helwlett- Packard 5890 series II plus, HP analytical Direct, Wilmington, DE) equipped with a flame ionization detector and a 30 m × 0.25 mm × 0.25 μm capillary column (Omega wax 250# 2–4136, Supelco). The helium gas flow rate was 1.2 ml/min with a split/flow ratio of 50:1. Oven temperature was held at 205°C. The injector and detector temperatures were 260 and 262°C, respectively. Two internal standards, C15:0 and C23:0, were added during the analysis. The fatty acids were identified by comparing the retention times with authentic standards [10].

### Statistical analysis

An SPSS computer program was used. The results are expressed as the mean ± S.D., and all statistical comparisons were made by means of independent t-tests, with P ≤ 0.001 being considered significant. Pearson correlations were also performed. Receiver Operating Characteristics analysis (ROC) was performed. The area under the curve, the cutoff values, and the degree of specificity and sensitivity were calculated. ROC curves are constructed by plotting the false positive rate (i.e. 100-specificity) against the true positive rate (i.e. sensitivity). These have been widely accepted as standard tools for evaluating the performance of biomarkers of toxicity. The AUC has been widely used as a quantitative index of biomarker performance in a variety of applied fields [11].

## Results

**Figure 1:**
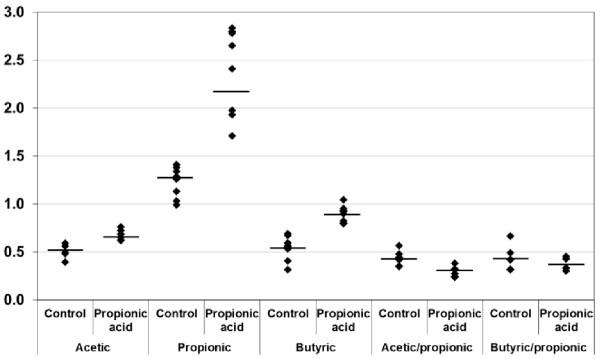
**Absolute and relative concentrations of SCFAs in control and PPA-intoxicated rats.** The mean value for each group is designated by a line.

**Figure 2:**
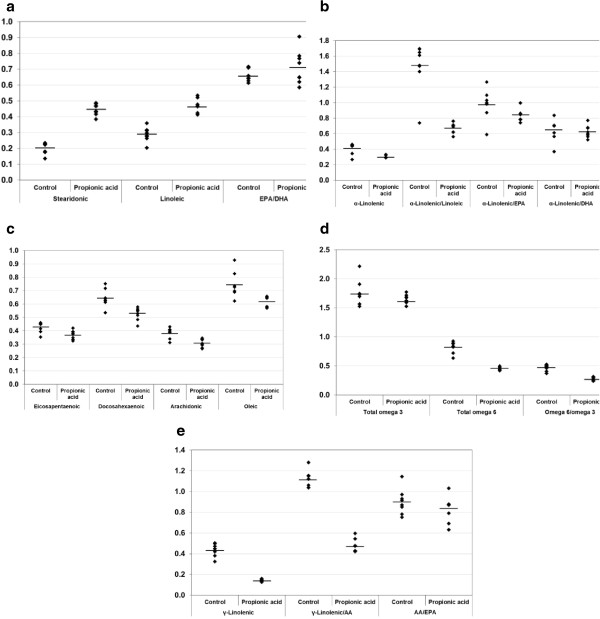
**(a-e) Absolute and relative concentrations of PUFAs in control and PPA-intoxicated groups.** The mean value for each group is designated by a line.

Data representing the absolute and relative values of SCFAs and PUFAs are presented as the mean ± S.D for 6 independent experiments in Tables 1 and 2. Figures 1 and 2 also present the mean values for each parameter from 6 independent experiments.

**Table 1:** **Mean ± S.D for the absolute (mmoles/gm brain tissue) and relative concentrations of propionic, acetic and butyric acids in the brain homogenates of the control and PPA-intoxicated rats**

Parameter	Group	N	Mean ± S.D.	Percent change	P value
Acetic	Control	8	0.524 ± 0.064	100.00	0.001
	Propionic acid	8	0.674 ± 0.049	128.58	
Propionic	Control	8	1.228 ± 0.159	100.00	0.001
	Propionic acid	8	2.385 ± 0.452	194.12	
Butyric	Control	8	0.540 ± 0.126	100.00	0.001
	Propionic acid	8	0.894 ± 0.086	165.70	
Acetic/propionic	Control	8	0.431 ± 0.069	100.00	0.001
	Propionic acid	8	0.291 ± 0.051	67.35	
Butyric/propionic	Control	8	0.433 ± 0.110	100.00	0.136
	Propionic acid	8	0.363 ± 0.062	83.67	

Table 1 describes the Independent Samples T-Test between the control and propionic acid groups for all parameters.

**Table 2:** **Absolute (nmoles/gm brain tissue) and relative concentrations of PUFAs in the Control and PPA- intoxicated rats**

Parameter	Group	N	Mean ± S.D.	Percent change	P value
α-Linolenic	Control	8	0.413 ± 0.070	100.00	0.004
	Propionic acid	8	0.310 ± 0.019	74.98	
Eicosapentaenoic	Control	8	0.428 ± 0.038	100.00	0.006
	Propionic acid	8	0.371 ± 0.033	86.70	
Docosahexaenoic	Control	8	0.644 ± 0.066	100.00	0.001
	Propionic acid	8	0.525 ± 0.047	81.60	
Linoleic	Control	8	0.288 ± 0.045	100.00	0.001
	Propionic acid	8	0.472 ± 0.042	163.78	
γ-Linolenic	Control	8	0.434 ± 0.060	100.00	0.001
	Propionic acid	8	0.144 ± 0.012	33.22	
Arachidonic	Control	8	0.385 ± 0.040	100.00	0.001
	Propionic acid	8	0.306 ± 0.030	79.44	
Total omega 3	Control	8	1.072 ± 0.228	100.00	0.001
	Propionic acid	8	0.819 ± 0.077	72.20	
Total omega 6	Control	8	0.896 ± 0.097	100.00	0.001
	Propionic acid	8	0.45 ± 0.033	83.3	
omega 6/Omega3	Control	8	0.836 ± 0.056	100.00	0.001
	Propionic acid	8	0.502 ± 0.026	100.27	
α-Linolenic/Linoleic	Control	8	1.466 ± 0.314	100.00	0.001
	Propionic acid	8	0.671 ± 0.066	45.78	
α-Linolenic/EPA	Control	8	0.978 ± 0.194	100.00	NS
	Propionic acid	8	0.831 ± 0.080	85.00	
α-Linolenic/DHA	Control	8	0.648 ± 0.138	100.00	NS
	Propionic acid	8	0.625 ± 0.079	96.49	
EPA/DHA	Control	8	0.665 ± 0.041	100.00	NS
	Propionic acid	8	0.712 ± 0.106	107.15	
ARA/Linoleic	Control	8	1.133 ± 0.081	100.00	0.001
	Propionic acid	8	0.644 ± 0.065	43.15	
ARA/EPA	Control	8	0.901 ± 0.123	100.00	NS
	Propionic acid	8	0.829 ± 0.124	91.98

It can be easily seen that SCFA, represented by propionic, butyric and acetic acid, was much higher in the brains of PPA-intoxicated rat pups compared with healthy controls. However, PUFAs and their precursors were significantly lower.

Table 1 also demonstrates a significantly lower acetic/propionic acid ratio of (0.291 ± 0.051) in treated rats compared with a higher ratio of (0.431 ± 0.069) in control untreated animals. A non-significant but still lower butyric/propionic acid ratio was recorded in intoxicated rats.

The relative values for PUFA, which are presented in Table 2, were substantially lower in the intoxicated rats compared with the control rats. Omega 6/ omega3, α-Linolenic/Linoleic, and AA/linoleic acid ratios were significantly lower in the PPA-intoxicated rats. The recorded values were 0.502 ± 0.026, 0.671 ± 0.066 and 0.644 ± 0.065, respectively, compared with the much higher values in the control animals of 0.836 ± 0.056, 1.466 ± 0.314, and 1.133 ± 0.081. Other ratios such as α-Linolenic/EPA, α-Linolenic/DHA, EPA/DHA and AA/EPA were not significantly different between the two groups.

Tables 3 and 4 demonstrate the ROC analysis of the absolute and relative ratios of the measured SCFAs and PUFAs. Satisfactory specificity and sensitivity values were recorded for most of these fatty acids.

**Table 3:** **ROC analysis represented as area under the curve (AUC), specificity and sensitivity for the measured SCFA in the PPA-intoxicated groups**

	Area under the curve	Best cutoff value	Sensitivity %	Specificity %
Acetic	1.000	0.603	100.0%	100.0%
Propionic	1.000	1.560	100.0%	100.0%
Butyric	1.000	0.740	100.0%	100.0%
Acetic/propionic	0.969	0.336	87.5%	100.0%
Butyric/propionic	0.625	0.375	62.5%	75.0%

**Table 4:** **ROC analysis represented as area under the curve (AUC), specificity and sensitivity for the measured PUFA in the PPA-intoxicated groups**

	Area under the curve	Best cutoff value	Sensitivity %	Specificity %
α-Linolenic	0.875	0.337	100.0%	87.5%
Eicosapentaenoic	0.883	0.405	87.5%	75.0%
Docosahexaenoic	0.938	0.595	100.0%	87.5%
Linoleic	1.000	0.386	100.0%	100.0%
γ-Linolenic	1.000	0.241	100.0%	100.0%
Arachidonic	0.938	0.338	87.5%	87.5%
Oleic	0.922	0.673	100.0%	87.5%
Total omega 3	0.609	1.690	75.0%	62.5%
Total omega 6	1.000	0.565	100.0%	100.0%
Omega6/omega 3	1.000	0.342	100.0%	100.0%
α-Linolenic/Linoleic	0.984	1.080	100.0%	87.5%
α-Linolenic/EPA	0.828	0.865	87.5%	87.5%
α-Linolenic/DHA	0.656	0.689	87.5%	62.5%
EPA/DHA	0.641	0.728	50.0%	100.0%
γ-Linolenic/ARA	1.000	0.817	100.0%	100.0%
ARA/EPA	0.625	0.896	87.5%	50.0%

Table 5 demonstrates Pearson correlations between the neurotoxin PPA and different absolute and relative concentrations of SCFAs and PUFAs.

**Table 5:** **Pearson correlations between the neurotoxin PPA and different measured absolute and relative concentrations of SCFAs and PUFAs**

Parameters	R (Pearson Correlations)	Sig.	
Propionic ~ Acetic	0.817^**^	0.001	P^a^
Propionic ~ Butyric	0.845^**^	0.001	P^a^
Propionic ~ Acetic/propionic	-0.892^**^	0.001	N^b^
Propionic ~ Butyric/propionic	-0.495	0.051	N^b^
Propionic ~ α-Linolenic	-0.604^*^	0.013	N^b^
Propionic ~ Stearidonic	0.888^**^	0.001	P^a^
Propionic ~ Eicosapentaenoic	-0.397	0.128	N^b^
Propionic ~ Docosahexaenoic	-0.561^*^	0.024	N^b^
Propionic ~ Linoleic	0.865^**^	0.001	P^a^
Propionic ~ γ-Linolenic	-0.826^**^	0.001	N^b^
Propionic ~ Arachidonic	-0.735^**^	0.001	N^b^
Propionic ~ Oleic	-0.530^*^	0.035	N^b^
Propionic ~ Total omega 3	-0.070	0.797	N^b^
Propionic ~ Total omega 6	-0.825^**^	0.001	N^b^
Propionic ~ Omega6/omega 3	-0.867^**^	0.001	N^b^
Propionic ~ α-Linolenic/Linoleic	-0.786^**^	0.001	N^b^
Propionic ~ α-Linolenic/EPA	-0.486	0.056	N^b^
Propionic ~ α-Linolenic/DHA	-0.022	0.935	N^b^
Propionic ~ EPA/DHA	0.317	0.232	P^a^
Propionic ~ γ-Linolenic/AA	-0.812^**^	0.001	N^b^
Propionic ~ AA/EPA	-0.468	0.067	N^b^

^a^Positive Correlation.

^b^Negative Correlation.

^*^Correlation is significant at the 0.05 level (2-tailed).

^**^Correlation is significant at the 0.01 level (2-tailed).

## Discussion

Understanding lipid and EFA metabolism is crucial for maintaining both physical and mental health. Working with the brain tissues of a rodent model orally intoxicated with PPA could offer new insight into brain disturbances related to the absolute and relative abundance of SCFAs and PUFAs and their relationship to different lipid mediators, such as leukotrienes and prostaglandins. It is well known that PGE_2_, which is an anti-inflammatory prostanoid, is induced by SCFAs. In addition to classical eicosanoids such as PGE_2_, other lipid mediators that include lipoxins, resolvins, protectins and maresins are also generated from PUFAs [12].

It is well known that high concentrations of butyrate prevent NF-kB activation in LPS-stimulated RAW264.7 murine macrophage cells [13]. In addition, other studies have reported that inhibition of NF-kB induced by butyrate in IFNγ-stimulated RAW264.7 is implicated in the butyrate-mediated reduction of TNFα, IL-6 and iNOS expression [14]. All of these actions, which are mediated by butyrate, may contribute to the anti-inflammatory effects of butyrate in inflammatory bowel disease (IBD), both in experimental models and in humans, where it has been reported that butyrate suppresses the activation of HSP70 and NF-kB [15, 16]. As dual effect of butyrate, brain microglial cells were found to be greatly affected. Sodium butyrate concentration of 0.6 mM but not that of 0.2 mM potentiated the LPS-induced secretion of both IL-6 and nitric oxide [17]. Most interestingly, butyrate treatment induced a proinflammatory response in transformed N9 microglial cell line and anti-inflammatory response in primary, brain-derived microglial cells. In contrast to the effect of butyrate on pro-inflammatory, anti-inflammatory cytokines show diverse pattern with dose-dependent dual effects. IL-10 as an important anti-inflammatory cytokine is increased by 0.25 mM concentration but significantly decreased by 1 mM of butyrate [18]. On the other hand, most of pro-inflammatory cytokines are significantly stimulated by high concentration of PPA [5]. A lower butyrate /propionate ratio was reported in the present study in the PPA-intoxicated group (Table 1 and Figure 1). This finding could be related and supported by our previous study in which pro-inflammatory markers (TNFα, INFγ, IL-6, and HSP70) are persistently induced in rat pups that are orally administered the same neurotoxic dose of PPA [6].

It is well known that acetic acid is utilized by hepatocytes and transformed into Acetyl-CoA, which can act as a precursor for lipogenesis and stimulate gluconeogenesis [19–21]. Propionic acid is mainly metabolized in the liver and has been shown to inhibit gluconeogenesis and increase glycolysis in rat hepatocytes [22]. It has also been proposed that propionic acid may lower plasma cholesterol concentrations by inhibiting hepatic cholesterogenesis [23]. The significantly lower acetic/propionic ratio reported in the present study (Table 1 and Figure 1) together with the associated depletion of glucose (a major energy source for brain cells) and cholesterol [22, 23] can identify PPA-induced neurotoxicity [6, 7]. Based on the fact that a deficiency in cholesterol and glucose greatly reduces glutamate uptake by glutamate transporters and leads to elevated glutamate, the reported lower acetic/PPA ratio could also be related to glutamate excitotoxicity, a mechanism that is strongly involved in the etiology of autism [20].

It is well documented that AA, EPA, and DHA are essential for the normal development and growth of the brain and of memory [24–27]. AA stimulates glucose uptake in cerebral cortical astrocytes; thus, it plays a critical role in the regulation of energy metabolism in the cerebral cortex [28]. AA and DHA enhance acetylcholine (ACh) release, which guarantees long-term synaptic plasticity, thereby improving learning ability in experimental animals [29]. Several clinical studies have shown that supplementing infants with AA, EPA, and DHA significantly improves their cognitive development and memory [30, 31].

Additionally, DHA promotes neuronal survival by facilitating membrane translocation/activation of Akt, which is a central player in signal transduction pathways and thus controls cellular functions such as proliferation and survival, metabolism, angiogenesis, and exocytosis. The in vivo reduction of DHA by dietary depletion increases the susceptibility of hippocampal neurons to apoptosis [32, 33].

Based on this information, the significant decrease in α LA, EPA, DHA, γ-LNA and AA reported in the present study can be easily related to PPA-neurotoxicity. Depletion of these PUFAs could indicate impairment of energy metabolism, loss of synaptic plasticity and increased neuronal susceptibility to apoptosis. These persistent biochemical autistic features were induced in rat pups that were orally administered the same neurotoxic dose of PPA [6]. The observed variation in the absolute and relative concentrations of FFA could be also related to the pro-inflammatory effects of PPA. While AA is considered a pro-inflammatory lipid, γ-LNA, EPA and DHA have been viewed as anti-inflammatory molecules due to their capacity to reduce the production of pro-inflammatory cytokines via the NF-κB signaling pathway [34, 35]. Moreover, EPA is itself a substrate for cyclooxygenase and lipoxygenase, giving rise to mediators that often have biological effects opposite to those of AA.

Linoleic acid (LA) is an essential dietary fatty acid that is crucial to neonatal development. It is necessary for the growth and development of the brain and other body tissues that are dependent on AA, the central n-6 eicosanoid precursor synthesized from LA via elongation-desaturation [36]. Desaturase and elongase enzymes insert double bonds and elongate carbon chains to create PUFA from essential fatty acid (EFA) precursors [37]. It appears that the same enzymes catalyze the conversion of both omega-6 and omega-3 fatty acid precursors into PUFAs. Lower desaturase activity, which is estimated by the ratio of AA to LA, might also be a neurotoxic effect of PPA. A 56.85% reduction in AA/LA could confirm the toxic effect of PPA on the brains of treated rat pups. A link between fatty acid desaturation genes and attention-deficits was previously suggested [38]. The non-significant change observed in αLA/EPA and αLA/DHA could be attributed to differences in Km values and affinity between the fatty acid desaturase enzyme and the substrates LA, ALA, EPA and DHA. This suggestion could be supported by the remarkable lower αLA/LA ratio, which are precursors for ω3 and ω 6 respectively.

Furthermore, PUFAs could be related to the neurochemical activity of the brain. These acids could control the expression, properties, and action of dopamine, serotonin (5-HT), and Ach [39, 40], especially during the perinatal period. During this period the growth and development of brain is at a maximum and ACh, in turn, regulates the release of PUFAs [41]. Thus, ω-3 PUFAs and AA modulate neural function, including neurotransmission, membrane fluidity, ion channel, enzyme regulation and gene expression and prevent inflammation. They could therefore be of significant benefit in the prevention and management of autism.

Fatty acids may exert structural effects on membranes, either as free entities (i.e. FFA) or as part of other molecules such as phospholipids and triacylglycerides. Omega-3 fatty acids have a curved shape, allowing gaps between molecules when they are incorporated into cell membranes. These gaps increase the fluidity of the membrane, enabling cell-to-cell communication with the aid of ion channels [42]. In contrast, omega-6 fatty acids are straighter and narrower and therefore reduce the fluidity of the membrane due to a remarkable decrease in the gaps between cells. Consequently, it is important that the ratio of the PUFA types remains balanced to preserve optimal functioning of the cell membrane [43].

The lower α-Linolenic acid and DHA reported in the present study (Table 2 and Figure 2) can also be related to impaired serotonin transmission, which was previously reported in rat pups either orally administered (the same toxic dose) or intraventricularly infused with PPA [6, 7]. α-Linolenic and DHA deficient rats exhibited significantly lower prefrontal cortex 5-HT content, greater 5-hydroxyindoleacetic acid 5-HIAA content, a significantly greater 5-HIAA/5-HT ratio, and a decrease in midbrain tryptophan hydroxylase-2 expression, which is a rate limiting enzyme in the 5-HT biosynthesis pathway. This finding suggests that a relationship exists between dysregulation in central 5-HT neurotransmission and omega-3 fatty acid deficiency [44].

In addition, ω -3 fatty acids may modulate biochemical and physiological responses that are implicated in neurodevelopment via their effects on nuclear transcription factors, especially those involved in immunologic dysfunction. In vitro neuronal cell studies have revealed DHA to be a potent ligand for peroxisome proliferator-activated receptor γ (PPAR γ), which results in a suppression of proinflammatory genes that encode various interleukins and tumor necrosis factor (TNF)-α [45].

The significantly lower ω 3, ω6 and ω 6/ω 3 ratios reported in the present study (Table 2 and Figure 2) are consistent with the previous work of Thomas et al. [46], in which PPA infusion in rats decreased the total levels of monounsaturates, ω 6 fatty acids, and phosphatidylethanolamine plasmalogens and decreased the ω 6/ω 3 ratio, providing evidence of a relationship between changes in brain lipid profiles and autism-like behaviors in a rodent model.

Changes in behavior and brain integrity in adult rodents following transient selective serotonin (5-HT) reuptake inhibitors (SSRIs) such as fluoxetine may be related to disturbed AA neurotransmission and metabolism because AA is released from synaptic membrane phospholipids during neurotransmission involving 5-HT2A/2C receptors [47–50]. As a second messenger, AA can modify multiple aspects of brain function and structure. It is a precursor of a large number of bioactive eicosanoid products within the brain’s AA metabolic cascade [51, 52].

Anti-inflammatory ω-3 derived EPA and DHA block sodium (Na^+^) channels in a dose and time dependent manner in neonatal rat ventricular myocytes [53, 54]. This in turn limits the activity of the Na^+^/Ca^+2^ exchanger, which regulates intracellular Ca^+2^ influx. In vivo cell culture studies demonstrate that both EPA and DHA increase cellular inactivation in the Cornu Ammonis area (CA1) region of the hippocampus, thus reducing Ca^+2^ influx-associated cellular activity [55]. Thus, PUFAs are likely to reduce intracellular Ca^+2^ influx-associated excitotoxicity.

In the present study, the impaired relative and absolute fatty acid levels could be related to mitochondrial dysfunction as an etiological mechanism in autism. PPA is thought to affect mitochondrial fatty acid metabolism by binding to propionyl Coenzyme A and by sequestering carnitine [56, 57]. This in turn induces potential metabolic disturbance by affecting mitochondrial β-oxidation and bioenergetics. On the other hand, impaired fatty acid oxidation activates uncoupling protein-2 (UCP-2), which results in heat generation that does not contribute to ATP production [58, 59].

The receiver operating characteristic (ROC) analysis (Tables 3 and 4) showed that most of the measured relative values for fatty acids can be used as biomarkers for PPA neurotoxicity, recording satisfactory specificity, sensitivity and AUC. While elevated propionic acid is significantly correlated with levels of acetic, butyric, stearidonic and linoleic acid, it is inversely associated with the rest of the absolute and relative values of PUFAs (Table 5). Omega 6 fatty acids, represented by γ-LA and AA, were more significantly related to PPA-neurotoxicity than ω-3 fatty acids (ALA, EPA, and DHA). γ-LA, AA, DHA and EPA all play key roles in brain function, especially via the synthesis of eicosanoids that have anti-inflammatory, anti-thrombotic, and vasodilatory properties [60]. Therefore, the inverse association between PPA level and the levels of these acids confirm that the neurotoxic dose of this short chain fatty acid used in this study (250 mg/kg body weight/day for three days) was effective in inducing an alteration in the brain fatty acid profile. These results provide evidence for a relationship between changes in brain lipid profiles and the occurrence of ASD-like biochemical alterations in a rodent autism model [6]. This can be easily supported by the positive associations between brain malondialdehyde (MD) and short chain fatty acids (PA and acetic acid), and the negative correlations between polyunsaturated fatty acids and MD as marker of increased oxidative stress, a status recently related to the aetiology and clinical presentation of autism [6, 61]. Another support can be easily found in our recent work in which impaired plasma phospholipids and relative amounts of essential polyunsaturated fatty acids were recorded as predictive biomarkers in Saudi patients with autism [62]. Based on this, we propose that altered absolute and relative amounts of fatty acids may contribute to ASD and can be used as biomarkers for early detection of neurotoxicity related to this disorder.
